# A Simulation Study of Categorizing Continuous Exposure Variables Measured with Error in Autism Research: Small Changes with Large Effects

**DOI:** 10.3390/ijerph120810198

**Published:** 2015-08-24

**Authors:** Karyn Heavner, Igor Burstyn

**Affiliations:** 1 Department of Environmental and Occupational Health, School of Public Health, Drexel University, Philadelphia, PA 19104, USA; E-Mail: Igor.Burstyn@drexel.edu; 2 A.J. Drexel Autism Institute, School of Public Health, Drexel University, Philadelphia, PA 19104, USA; 3 Department of Epidemiology and Biostatistics, School of Public Health, Drexel University, Philadelphia, PA 19104, USA

**Keywords:** categorization, autism spectrum disorders, epidemiology methods, misclassification, dichotomization

## Abstract

Variation in the odds ratio (OR) resulting from selection of cutoffs for categorizing continuous variables is rarely discussed. We present results for the effect of varying cutoffs used to categorize a mismeasured exposure in a simulated population in the context of autism spectrum disorders research. Simulated cohorts were created with three distinct exposure-outcome curves and three measurement error variances for the exposure. ORs were calculated using logistic regression for 61 cutoffs (mean ± 3 standard deviations) used to dichotomize the observed exposure. ORs were calculated for five categories with a wide range for the cutoffs. For each scenario and cutoff, the OR, sensitivity, and specificity were calculated. The three exposure-outcome relationships had distinctly shaped OR (*versus* cutoff) curves, but increasing measurement error obscured the shape. At extreme cutoffs, there was non-monotonic oscillation in the ORs that cannot be attributed to “small numbers.” Exposure misclassification following categorization of the mismeasured exposure was differential, as predicted by theory. Sensitivity was higher among cases and specificity among controls. Cutoffs chosen for categorizing continuous variables can have profound effects on study results. When measurement error is not too great, the shape of the OR curve may provide insight into the true shape of the exposure-disease relationship.

## 1. Introduction

The cutoff used to dichotomize a continuous exposure variable may have a profound effect on the measures of association and interpretation of study results [1,2]. It is well-understood that, as the threshold for definition of “exposure” changes, the magnitude of the effect estimates, such as odds ratio (OR), will vary as well even though the true relative risk per unit of exposure is constant. For example, if one studies the association between obesity measured as body-mass index (BMI) and myocardial infarction, and categorizes BMI, then if (a) we set a threshold at BMI = 25 then we compare “underweight” and “overweight”, but if we (b) set a threshold at BMI = 30, then we compare “obese” patients with the rest. It is reasonable to expect different estimates of risk in the two analytical approaches even though they arise from the same underlying association of obesity and myocardial infarction. Frequently, data are analyzed using multiple cutoffs but only one of these categorizations is reported, creating publication bias *in situ* [3]. Presenting results for a wide range of plausible cutoffs, by plotting effect estimates against cutoffs, minimizes the potential for fishing for particular results, makes study results more useful to readers, and avoids the pitfalls of using data driven cutoffs or theory or guidance driven cutoffs that may change [4]. Analyzing continuous exposure data using more than one cutoff (two categories) magnifies the problem of selecting cutoffs as many different combinations of cutoffs may be plausible and estimates become increasingly unstable in finite datasets as the number of categories increases [5]. However, it is routinely done, with some justification, to explore the shapes of exposure-response associations, although even this has been criticized, with alternatives involving non-parametric techniques being suggested [6,7]. In addition, if more than two categories are analyzed, the cutoffs are frequently data driven, which has its own limitations.

Environmental exposures and gestational age are examples of exposures that are inherently continuous but are frequently categorized and for which the recommended cutoffs tend to change over time or are under debate. For example, environmental regulations tend to lower the exposure threshold at which a change in risk is of interest, rendering studies presented with a limited range of cutoffs (perhaps based on exposure limits in existence at the time of the study) less useful for future risk assessment or meta-analysis. Likewise, the clinical thresholds for shortened gestation and “term” pregnancy have evolved over time [8,9] and underscore the importance of investigating more than one cutoff for gestational age. Our previous research presented estimates of effect for a wide range of cutoffs used to dichotomize the exposure of interest on an OR curve, plotting OR *versus* the cutoff [1,4]. An “OR curve” was used in the context of autism spectrum disorders (ASD) research with results presented for a range of gestational age cutoffs to help clarify heterogeneity of the ASD-gestational age associations reported in the literature [4]. Our previous work presented OR curves for associations for which the shape of the underlying association was unknown.

There are also methodological caveats with dichotomization of an exposure that is imperfectly measured [10,11]. Even when measurement error in a continuous exposure is non-differential before categorization, the process of categorization creates dependence of the resulting misclassified binary variable on the outcome when (1) exposure categories are created from a continuous exposure that is assessed with error and (2) the exposure and outcome are not independent, producing heterogeneity of risk within exposure categories [12,13]. This study is an investigation of the first condition. In practice one can never be sure of the second condition (independence of exposure and outcome), and therefore evaluation of the degree of association must consider the possibility of differential misclassification. However, if one was certain that exposure and outcome are independent, then there would be no need to investigate their association using any exposure metric and the study investigating their association would not take place, rendering condition 2 of no import here.

The extent to which such sources of differential misclassification produce important biases in epidemiologic analyses is currently not known as it can be expected to depend on multiple factors, including the degree of measurement error, the true strength of the effect of the exposure on the outcome, and the choice of the cutoff value. The contribution of model misspecification to bias in studies that categorize a mismeasured exposure variable is not known and it is beyond the scope of the present study to gauge the relative importance of the various sources of error. The influence of the true shape on the exposure-outcome association biases that arise from dichotomization is currently unknown as all prior research was conducted for “linear” models (in a sense that log (relative risk) was linear with exposure in simulations). It has long been recognized that this is a topic of some concern (e.g., [13]) but comprehensive evaluation of the degree of non-differential misclassification created by dichotomization is lacking.

In this study we investigated the effects of changing the cutoffs for a hypothetical causal exposure, assessed with and without measurement error, on the association with ASD in a simulated population similar to the source population for the Early Autism Risk Longitudinal Investigation (EARLI). We also build on our previous work (in which the true shape of the underlying association was unknown) by investigating whether the shape of the OR curve provides insight into the true underlying shape of the exposure-outcome relationship. EARLI is an enriched-risk pregnancy cohort (mothers of a child affected by an ASD enrolled at the start of a subsequent pregnancy) intended to investigate environmental exposures potentially important in the etiology of ASD (http://www.earlistudy.org/) [14]. Our previous work that was informed by the EARLI described the potential impact of measurement error in exposures and outcomes under different scenarios of study size, precision of exposure measurement and magnitude of the true association [15]. Importantly, numerous continuous environmental exposures for which ideal cutoffs are either unknown (e.g., brominates, flame retardants, bisphenol-A) or contentious (e.g., mercury and lead) are of interest to EARLI due to suspicion of their neurotoxicity that is relevant to ASD. Although our work is motivated by EARLI, the simulations we conduct and conclusions should be of general interest; keeping the simulations closely connected to EARLI not only serves our own interest, but also illustrates how our simulation study can be adapted to the complexities of a specific problem rather than presenting conclusions for some unrealistically simplistic set-up.

**Table 1:** True and observed values in the simulated population.

Values in the Population (Notation)	True Values	Observed Values	Measurement Error	Postulated True Association with Latent Measure of Outcome ^a^	Cutoffs Range (Increments)
Environmental exposure 1 (X_1_)	X_1_ ~ N(0,1), correlated with X_2_ by Pearson correlation ρ = 0.7	W_1_ = X_1_ + ε_1_	ε_1_ ~ N(0, σ^2^), where σ^2^ ℘ {0.0625, 0.25, 1}	{0.15, 0.25, 0.5}	−3 to 3 (in increments of 0.1) See Figure 1 for cutoffs for 5 categories
Environmental exposure 2 (X_2_)	X_2_ ~ N(0,1), correlated with X_1_ by Pearson correlation ρ = 0.7	W_2_ = X_2_ + ε_2_	ε_2_~N(0, 0.25)	0	<1 *vs*. ≥1
Sex (Z)	Z~Binomial(0.5, 1)	Z	None	1	
Gestational age (X_ga_)	X_ga_ = (43 – γ), where γ ~ χ^2^(3) 1 week was subtracted from the above gestational age for 5% of males	W_ga_ = R((Xga + ε_ga_); 23, 43), Where R(.) ^b^ is function that round expression to integers, and then truncated to 23 to 43 weeks.	${\text{ε}}_{\text{ga}}\;\sim \text{ N}(0,\;\frac{1}{7^{2}})$	0.1	<37 *vs*. ≥37
Autism endophenotype (latent, Y)	Linear model: Y_Linear_ = β_1_X_1_ + β_2_X_2_ + β_3_Z + β_4_X_ga_ + ε_y_ “Threshold” (semi-linear) model: If x_1_ < mean_x1_-standard deviation_x1_ then Y_Threshold_ = β_2_X_2_ + β_3_Z + β_4_X_ga_ + ε_y_ If x_1_ ≥ mean_x1_-standard deviation_x1_ then Y_Threshold_ = 1.5 × β_1_X_1_ + β_2_X_2_ + β_3_Z + β_4_X_ga_ + ε_y_ “Saturation” (semi-linear) model: If x_1_ < mean_x1_-standard deviation_x1_ then Y_Saturation_ = 1.5 × β_1_X_1_ + β_2_X_2_ + β_3_Z + β_4_X_ga_ + ε_y_ If x_1_ ≥ mean_x1_-standard deviation_x1_ then Y_Saturation_ = 0.5 × β_1_X_1_ + β_2_X_2_ + β_3_Z + β_4_X_ga_ + ε_y_, ε_y_ ~ N(0,1)	Y ^b^ = R(T(y); 0, 18), where T(.) is a function that is transformed to a Y log-normal distribution that matched the observed AOSI in EARLI	due to rounding by R(.) ^b^	Not applicable	0–6, 7–18

**^a^** coefficients of linear regression, see text and bottom of the table for details, β’s; ^b^ R(f(.); min, max) is the function that rounds values of function f(.) to integers and truncates values (retains only values) that fall within interval [min, max].

## 2. Materials and Methods

### 2.1. Simulated Population

The population parameters used in this and our previous work [15] are listed in Table 1. Briefly, a population cohort of 1,000,000 children was generated with observed sex (Z) and gestational age (W_ga_) distributions similar to the general US population. We generated such a large simulated dataset in order to evaluate how our method performs when instabilities due to sampling error and small strata are not an issue. We simulated true gestational age X_ga_ (in weeks) in the cohort as 43 − γ where γ ~ ~χ^2^(3) distribution, after which 5% of boys were assigned gestational age that was 1 week shorter to account for shortened gestation, on average, among boys [16]. Completed weeks of gestational age were considered (as is typically reported) and only live births between 23 and 43 weeks (inclusive) gestational age were included in the simulation.

The cohort had simulated exposure to two agents: exposure 1, represented by X_1_, and exposure 2, represented by X_2_,that both follow standard normal distributions and are correlated (Pearson ρ = 0.7). Only exposure 1 exerts causal influence on ASD-related phenotype. This constellation of covariates assumed that there is no true confounding, since the outcome is independent of X_2_ and all other covariates are independent. The simulated population was created and analyses of the entire population (not a sample thereof) were conducted using SAS version 9.2 (SAS Institute, Cary, NC, USA). Ethics committee review and informed consent were not necessary as this was a simulation study. Our SAS code will be made available to other researchers upon request.

### 2.2. Covariate Measurement Error

We assumed that the two environmental exposures are observed with classical measurement error. The first (W_1_ = X_1_ + ε_1,_ ε_1_ ~ N(0,σ^2^), where σ^2^ ∈ {0.0625, 0.25, 1} and will be referred to as the measurement error variance) is a causal exposure. The extent of measurement error spanned the plausible range: From good precision of environmental measurements with error approximately 6% of true exposure variability to poor precision with error variance equal to the true exposure variability. We present in detail only results for σ^2^ of 0.25 and 1, with the full details given in the Appendices. The second exposure (W_2_) was not causal (W_2_ = X_2_ + ε_2,_ where ε_2_ ~ N(0,0.25)). Gestational age was subject to two sources of error: (a) Classical error in the observed continuous measure of the length of gestation, W_ga_ = X_ga_ + ε_ga,_ where ε_ga_ ~ N(0,σ^2^_ga_) and (b) round-off error as gestational age was reported in completed weeks (23−43 weeks). All errors were conditionally independent of each other.

### 2.3. Linear and Semi-Linear Risk Models

In addition to X_1_, sex (sex ratio of 4 males: 1 female [17]) and gestational age [18,19,20] exerted some causal influence on dichotomous ASD status and continuous ASD-related phenotype (Y). We verified that these yielded parameter estimates within the expected range. The true shape of the exposure-response curve is unknown so the simulated disease was modeled using the following three forms:

Linear model:
  Y_Linear_ = β_1_X_1_ + β_2_X_2_ + β_3_Z + β_4_X_ga_ + ε_y_
“Threshold” (semi-linear) model:
  If x_1_ < mean_x1_-standard deviation_x1_ then Y_Threshold_ = β_2_X_2_ + β_3_Z + β_4_X_ga_ + ε_y_
  If x_1_ ≥ mean_x1_-standard deviation_x1_ then Y_Threshold_ = 1.5 × β_1_X_1_ + β_2_X_2_ + β_3_Z + β_4_X_ga_ + ε_y_
“Saturation” (semi-linear) model:
  If x_1_ < mean_x1_-standard deviation_x1_ then Y_Saturation_ = 1.5 × β_1_X_1_ + β_2_X_2_ + β_3_Z + β_4_X_ga_ + ε_y_
  If x_1_ ≥ mean_x1_-standard deviation_x1_ then Y_Saturation_ = 0.5 × β_1_X_1_ + β_2_X_2_ + β_3_Z + β_4_X_ga_ + ε_y_,

The slope of the causal association with X_1_ above and below the inflection point defined by (mean_x1_ − standard deviation_x1_) was altered to create deviation from linearity. For example, in the saturation model, the effect of X_1_ is three times greater below than above the inflection point (1.5 × β_1_X_1_ / 0.5 × β_1_X_1_ = 3). These multipliers were chosen arbitrarily but have numerical values that yield plausible “average” associations if the linear model was fitted to the data. For all models ε_y_ ~ N(0,1), β_2_ was 0 since X_2_ had no true effect on Y. The latent values of ASD-related phenotype (Y_Linear, Threshold, Saturation_) were computed for each of three β_1_ ∈ {0.15, 0.25, 0.5} that correspond to “weak”, “moderate”, and “strong” underlying associations (see Heavner *et al*., [15] for more details on the simulation parameters). We present results only for the weak and strong associations in text, with all the details available in the Appendices.

### 2.4. Case Definition and Outcome Misclassification

The values of Y_Linear_, Y_Threshold_, and Y_Saturation_ were transformed to yield values in the valid range (0–18) for Autism Observation Scale in Infants (AOSI) scores [21], the outcome measure of interest. Latent continuous ASD-related endophenotype, was subjected to rounding error and categorized using a single clinically relevant cutoff. For Y_Linear_, Y_Threshold_ and Y_Saturation_, $Y_{c}^{*}=0$ if Y^*^ is 0–6 and $Y_{c}^{*}=1$ if Y^*^ is 7–18 (Table 1). For all exposure-disease models, subjects with a high AOSI score were considered to be cases. An AOSI score of at least seven has been shown to have clinical and/or etiologic significance. It must be noted that we do not equate high AOSI with a clinical diagnosis of ASD. We merely assert that ASD is a collection of traits that naturally occur on a continuous scale and are segregated into binary disease and healthy groups based on some criteria. This is consistent with the current conceptualization of ASD as a “spectrum” of phenotypes, not a definitive state common to all cases, as is true of other conditions (e.g., death from a cardiac event or acquisition of an infection). Various AOSI sub-scales may be more related to environmental exposure than others but this is not our focus here and our argument applies to any measures of continuous traits that have AOSI-like properties.

### 2.5. Investigating the Effect of Changing the Cutoff(s) for the Observed Causal Exposure (W_1_)

The mismeasured exposure was dichotomized in the first set of analysis and then a separate analysis was conducted where it was divided into five categories.

Two Categories

W_1_ was *dichotomized*, yielding W_1c_, at 61 different cutoffs (cutoff = φ) ranging from the population mean plus or minus three standard deviations in increments of 0.1 such that W_1c_ = 1 if W_1_ ≥ φ, and W_1c_ = 0 if W_1_ < φ. The reference group for these dichotomized variables was W_1c_ = 0.

Five Categories

W_1_ was then divided into *five categories* based on the number of standard deviations away from the mean value in the cohort. Four cutoffs (φ_1_ – φ_4_) were used such that the reference group was between 

**Figure 1:**
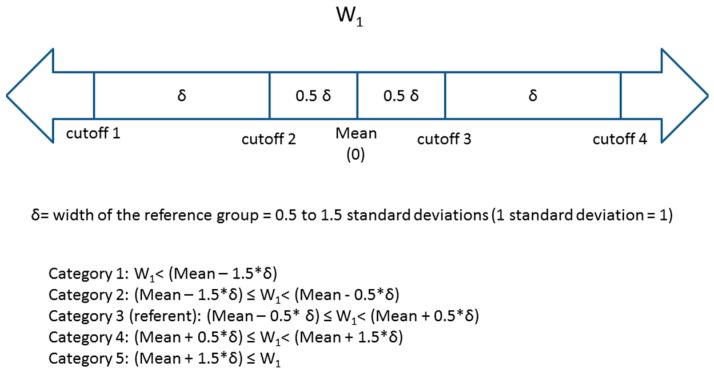
Descriptions of four cutoffs and five categories for W_1_.

φ_2_ and φ_3_ (referent group: φ_2_ ≤ W_1_ < φ_3_) (Figure 1). The distance between two consecutive cutoffs varied (distance between cutoffs = width of the reference group = δ) from 0.5 standard deviations to 1.5 standard deviations. The midpoint of category 3 (the reference category) was the estimated population mean (0) of W_1_. This resulted in three groups of equal width δ (the 2nd, 3rd (reference) and 4th groups) and two groups of varying width at the extremes of the exposure distribution (groups 1 and 5). A number of other multi-category approaches are reasonable and used in epidemiology (e.g., categories formed to ensure equal number of “cases”, categories reflecting percentiles of exposure distribution in disease-free individuals, categories based on hypothesized thresholds or other quantities with intuitive interpretation such as those relevance to clinical practice) but it is beyond the scope of this paper to compare them. We present an approach that bases the categories on the distribution of the exposure in the entire cohort; we make our SAS code available upon request for other researchers to explore alternative scenarios.

### 2.6. Assessing the Effect of Variation in the Cutoff for W_1_

For each W_1c_, a logistic model was computed in the form:

$$LOGIT\left(pr\left(Y_{c}^{*}=1\right)\right)=c_{0}+c_{1}W_{1c}+c_{2}W_{2c}+c_{3}Z+c_{4}W_{gac}\;(logistic).$$

Thus, for logistic models, exp (c_1_) is the odds ratio of the estimated effect of W_1c_ (OR_1_) on odds of AOSI ≥ 7. All independent variables were considered as dichotomized predictors (W_2c_ = 1 if W_2_ ≥ 1, W_2c_ = 0 if W_2_ < 1 and W_gac_ = 1 if W_ga_ ≥ 37, W_gac_ = 0 if W_ga_ < 37) of the dichotomized AOSI score. Although these variables are not true confounders, their inclusion in the logistic models realistically simulates regression analyses that are common in ASD research. In addition, the inclusion of these variables is not detrimental to the model from a statistical perspective as the size of the population is very large. We also fitted analogous logistic regression models with values of the exposures that were not contaminated by measurement error, e.g., with X_1_ instead of W_1_, *etc*. These models were repeated for the threshold and saturation models.

OR curves were created for the analyses of changing the cutoff used to dichotomize W_1_. These curves were created by plotting the OR_1_ derived from logistic regression on the y-axis and the cutoff used to dichotomize W_1_ (φ) on the x-axis. A separate set of plots of the ORs *versus* the width of the reference group (δ) was created for the scenarios with five categories of W_1_ (yielding points for four ORs for each width of the reference group).

We examined the degree of misclassification and its dependence on the outcome created by dichotomization of W_1_. The true classification with respect to the cutoff was the model without measurement error, *i.e.*, when X_1_ is dichotomized. For each cutoff of W_1_, the sensitivity and specificity were calculated separately for cases and controls. Sensitivity and specificity were defined as follows:

$$Sensitivity=\frac{\#\;exposed\;both\;without\;measurement\;error\;and\;with\;measurement\;error}{\#\;exposed\;without\;measurement\;error}$$

$$Specificity=\frac{\#\;unexposed\;both\;without\;measurement\;error\;and\;with\;measurement\;error}{\#\;unexposed\;without\;measurement\;error}$$

## 3. Results and Discussion

**Figure 2:**
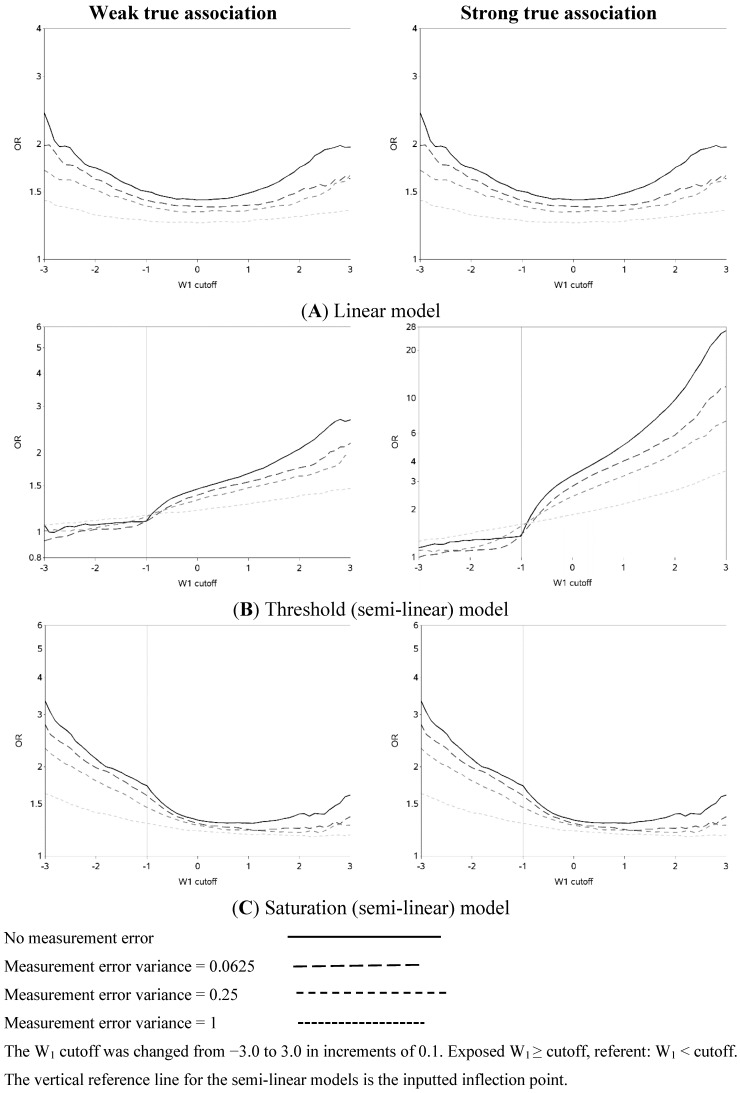
Odds ratios (ORs) for different cutoffs (between mean ± 3 standard deviations) used to dichotomize a mismeasured exposure (W_1_).

**Figure A1:**
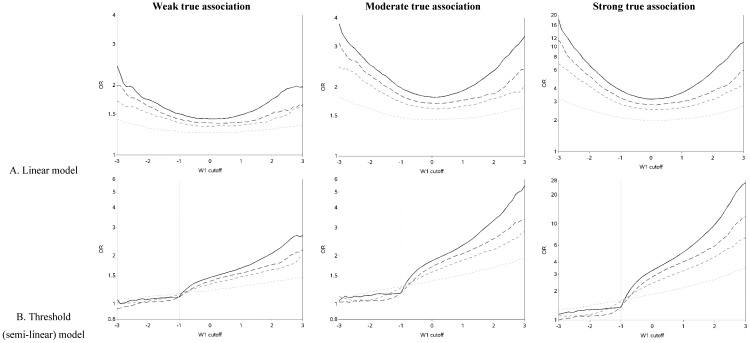

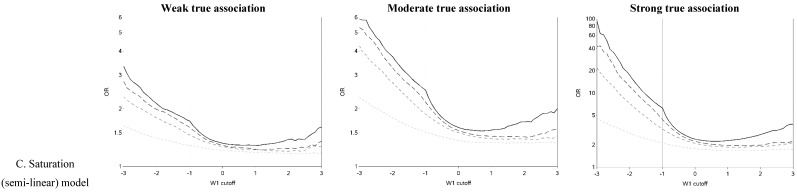
Odds ratios for different cutoffs (between mean ±3 standard deviations) used to dichotomize a mismeasured exposure (W_1_) (all scenarios for Figure 2). The X_1_ cutoff was changed from −3.0 to 3.0 in increments of 0.1. Exposed W_1_ ≥ cutoff, referent: W_1_ < cutoff. The vertical reference line for the semi-linear models is the inputted inflection point.

The characteristics of the population are similar to what was expected based on the simulation parameters (data not shown). The effect of changing the cutoff for dichotomizing W_1_ is shown in Figure 2 (and Figure A1). Each of the three exposure-outcome relationships had a distinctly shaped OR curve. Even with such a large population (n = 1,000,000), at the extreme cutoffs, there was non-monotonic oscillation in the effect estimates that is open to various interpretations. (In all scenarios, the greatest cutoff for the mismeasured exposure yielded more than 750 exposed cases, which is rarely attained even in ‘large’ epidemiology studies. For example, in the linear model with a weak true association and small measurement error, there were 846 cases with a mismeasured exposure at least as great as the highest cutoff.) The linear models had U-shaped graphs with slightly lower ORs for very large cutoffs compared to very small ones. For all strengths of the true association, the OR was increasingly underestimated (compared to the scenario with no measurement error) and the OR curve became flatter as measurement error increased. The highest estimates of OR tend to occur when cutoffs are made at the extremes of the exposure distribution, with changes from an easily dismissed OR of <2 tending to occur with the cutoff in the center of the distribution and a clear “signal” of OR >2 apparent with categorizations at the extremes when there was a moderate underlying association.

The semi-linear (threshold and saturation) models had a true inflection point (at one standard deviation below the mean). This inputted inflection point was obvious in the OR curves constructed under the semi-linear models without measurement error (Figure 2B,C). The OR curves appeared more linear and the inflection point was more obscured as the measurement error variance increased. In general, the OR became closer to the null as the measurement error increased. It is noteworthy that for the threshold model, contrary to intuition, dichotomization near the inflection point results in overestimation, rather than attenuation, of the OR in the presence of measurement error.

The two distinct slopes (below and above the mean minus one standard deviation) were clear for both the threshold and saturation models without measurement error. There was an apparent inflection point for the threshold models with moderate or large variance in the measurement error. The shape of the graph changed and the inflection point seemed to move as the measurement error variance increased. The shape of the curves for the threshold model for cutoffs greater than the mean resemble the steeper curves of the equivalent part of the curves for the linear models, as expected. It is also apparent that the highest ORs are obtained when the cutoff is made in the steeper section of the true exposure-response curve: this can be used to distinguish the linear from non-linear exposure-response associations. The influence of choice of cutoff on the overall results is perhaps most clear in the case of the saturation model: for a moderate true association, cutoffs below the inflection point tend to yield evidence of a “strong” association (OR > 3) while cutoffs above the inflection point yield an OR less than two. ORs less than two are frequently dismissed as unimportant in the epidemiology literature. 

**Figure 3:**
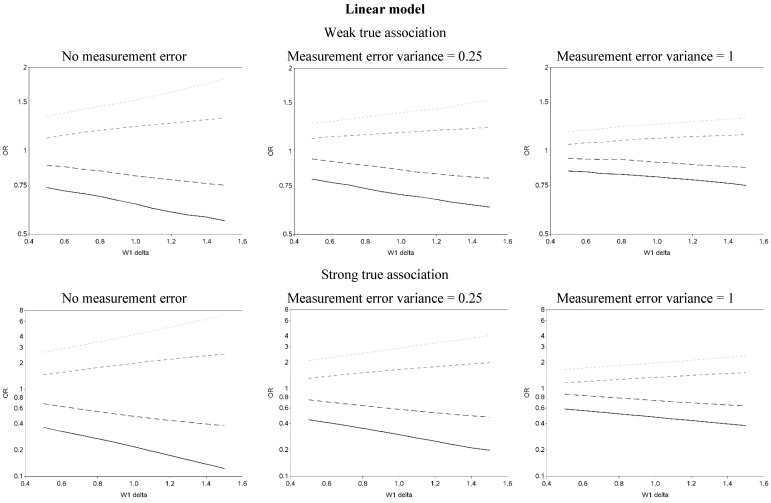

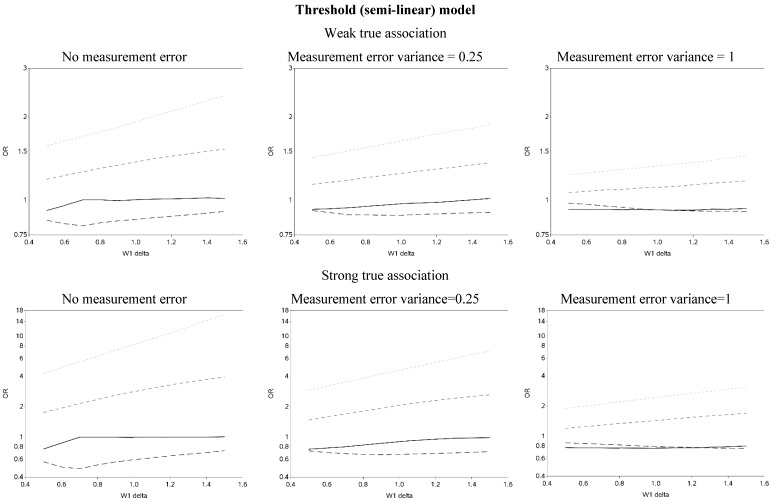

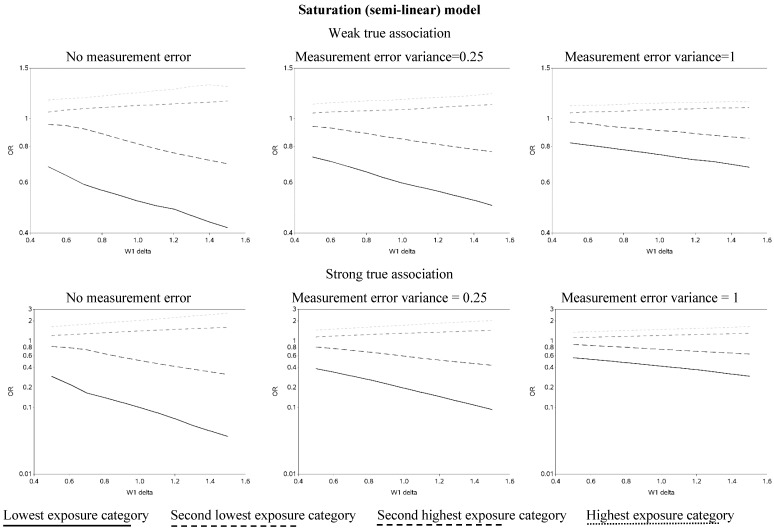
Odds ratios (OR) for different cutoffs used to create 5 categories for the causal yet mismeasured exposure (W_1_) (δ is identified as “W1 delta” in the figure). The 4 W1 cutoffs were centered at 0 with δ from 0.5 to 1.5 standard deviations, in increments of 0.1. Reference category for W1 is mean +/− (0.5 × δ × standard deviation).

**Figure A2:**
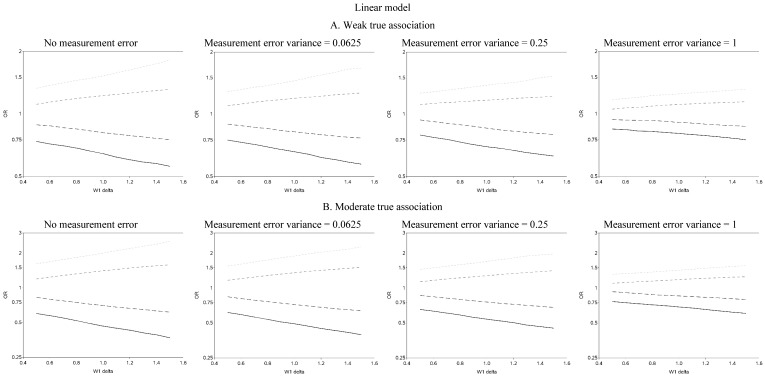

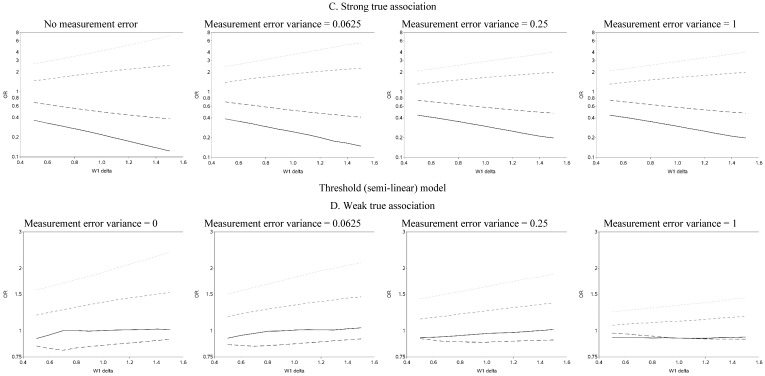

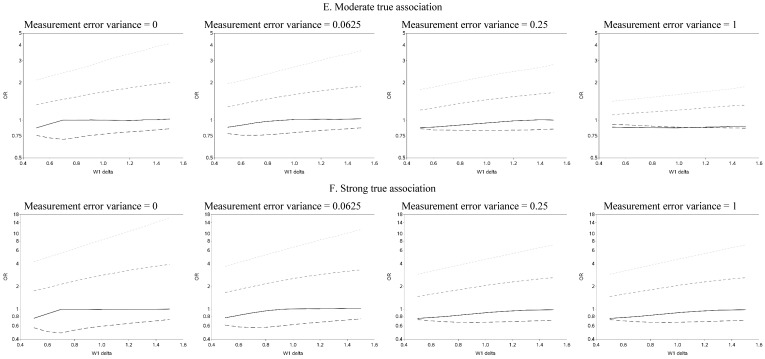

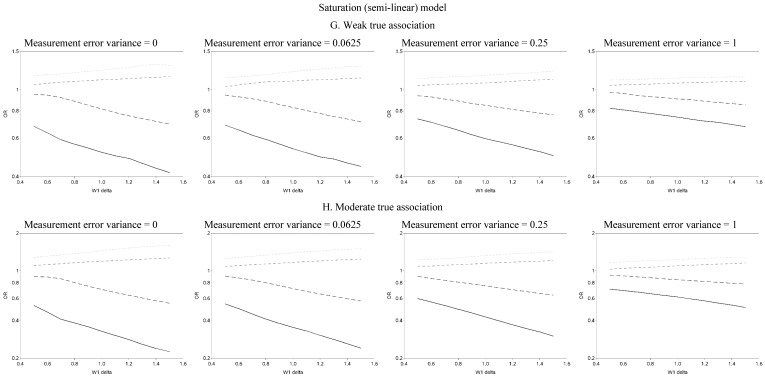

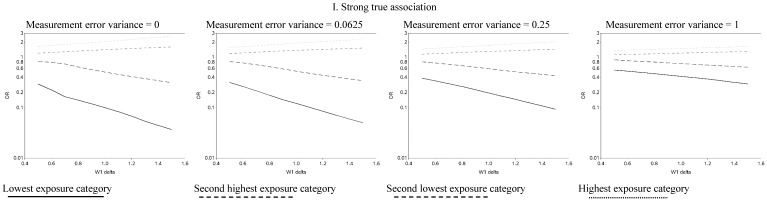
Odds ratios (OR) for different cutoffs used to create 5 categories for the causal yet mismeasuered exposure (W_1_) (all scenarios for Figure 3) (δ is identified as “W1 delta” in the figure). The 4 W1 cutoffs were centered at 0 with δ from 0.5 to 1.5 standard deviations, in increments of 0.1. Reference category for W1 is mean +/− (0.5 × δ × standard deviation).

The effect of changing the cutoffs used to create five categories of the causal exposure is illustrated in Figure 3 and Figure A2. The graphs were distinct for each of the three exposure-response models. For the linear models, as the width of the reference group (δ) increased, the ORs were farther from the null for all scenarios. In addition, as the measurement error variance increased, the ORs for all four categories moved closer to the null. Again, we note that whereas under low measurement error the graphs can be used to distinguish between associations of different shapes, this identification of shapes becomes problematic as measurement error increases, even with the increase in number of categories from two to five. In practice, this may lead to both under- and over-interpretation of information contained in the data about the shape of the exposure-response relationship (e.g., dismissing non-linearity when it is present, or positing a curvilinear shape when the exposure-response is linear, respectively), even though it would leave little doubt that there is an association between the exposure and outcome.

**Figure A3:**
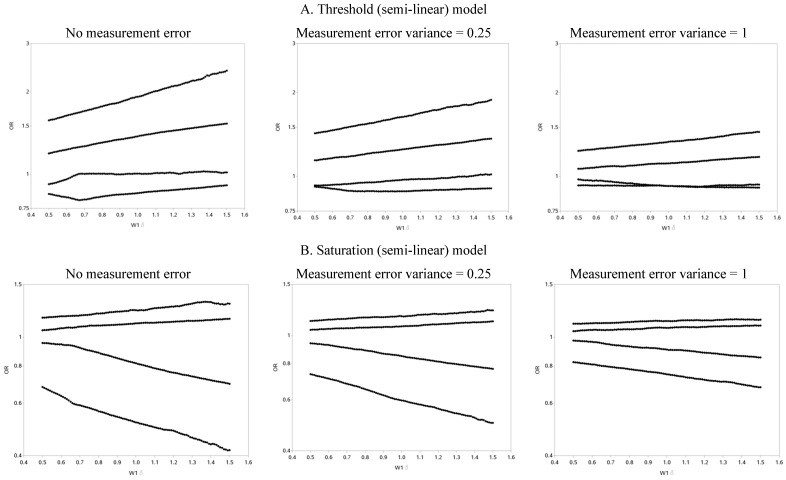
Odds ratios (OR) for different cutoffs used to create 5 categories for mismeasured exposure (W_1_), 101 cutoffs, with the true “weak” association. The 4 W1 cutoffs were centered at 0 with δ from 0.5 to 1.5 standard deviations, in increments of 0.1. Reference category for W1 is mean +/−(0.5 × δ × standard deviation).

When the distance between cutoffs was shortened it was possible to gain more insight into possible thresholds and inflection points (Figure A3 contains OR curves when the true association was weak, varying the cutoffs used to create five categories by smaller increments (0.01)). 

**Figure 4:**
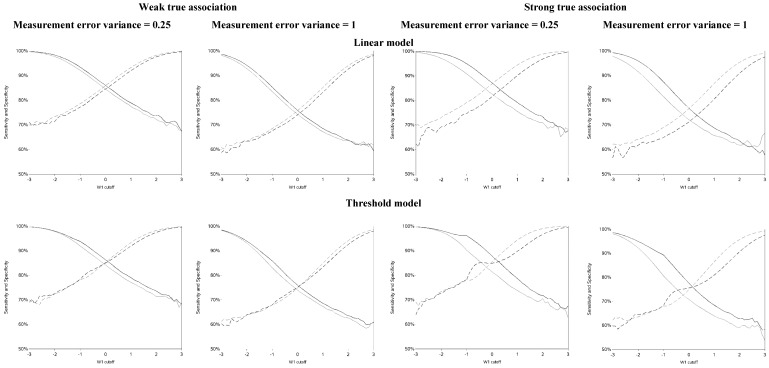

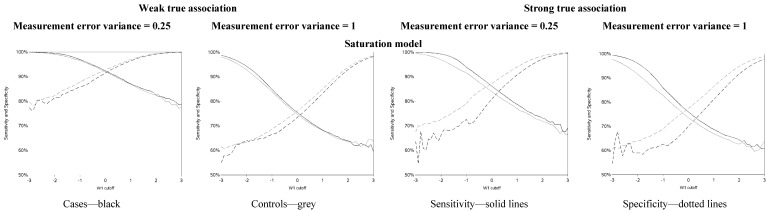
Sensitivity and specificity at each cutoff for the mismeasured exposure (W_1_).

**Figure A4:**
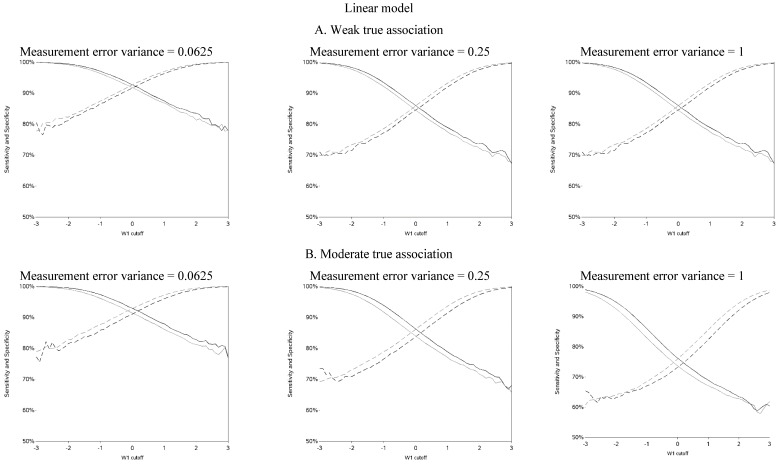

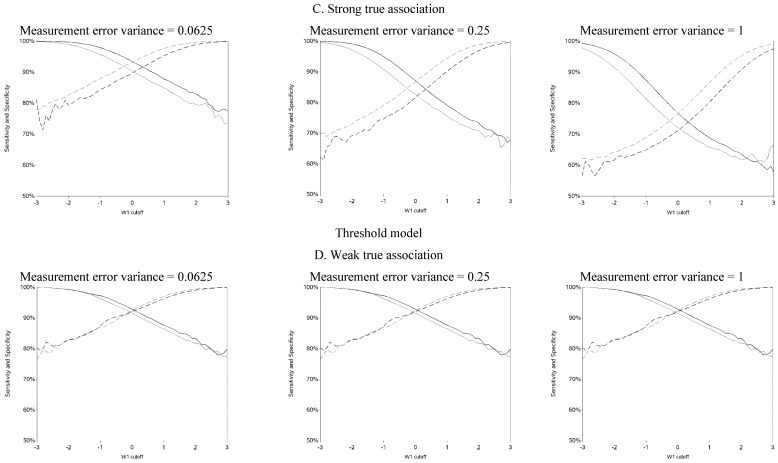

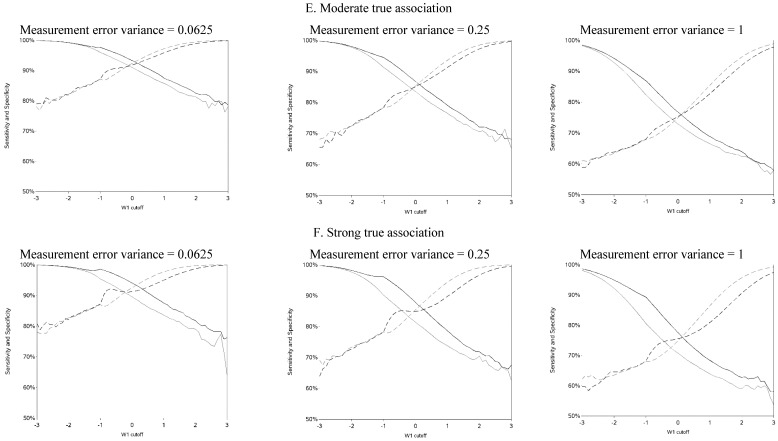

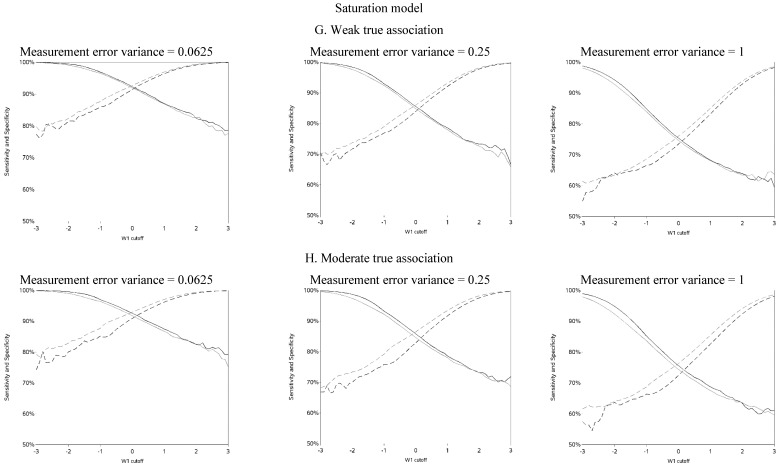

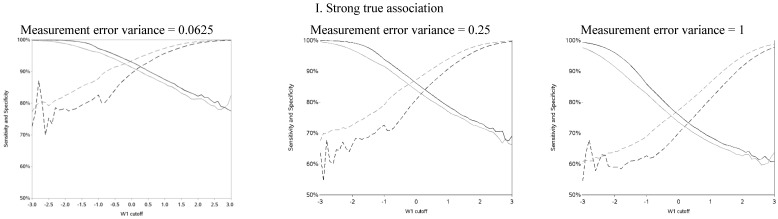
Sensitivity and specificity at each cutoff for W_1_ (all scenarios for Figure 4). Cases—black; Controls—grey; Sensitivity—solid lines; Specificity—dotted lines.

**Figure A5:**
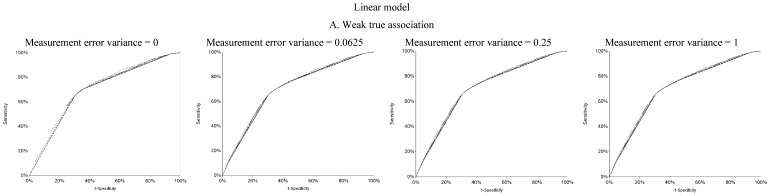

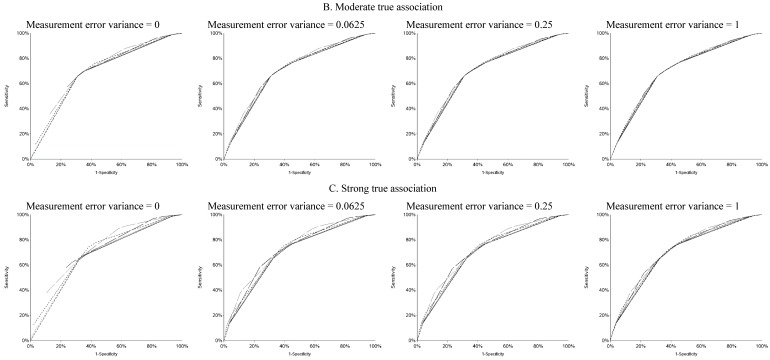

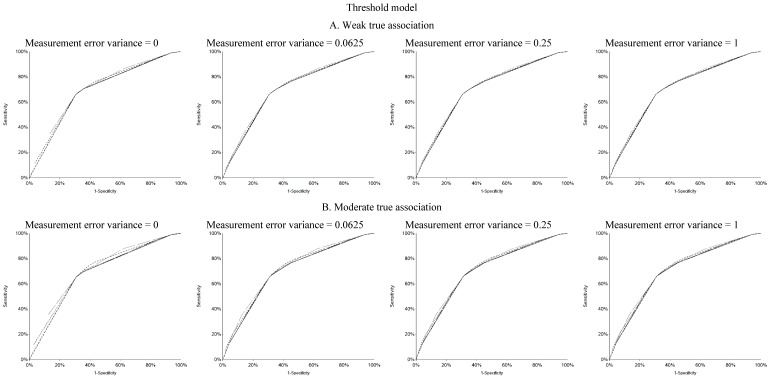

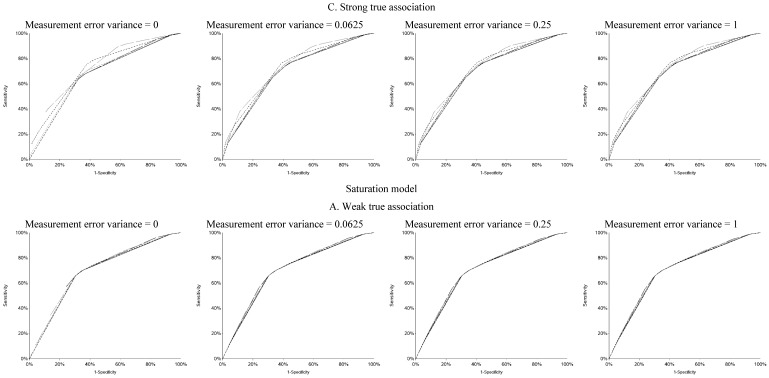

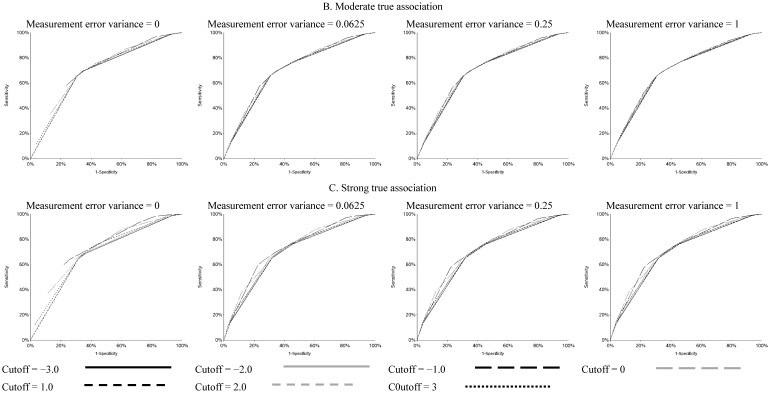
ROC curves for selected cutoffs for W_1_.

The sensitivity and specificity for each cutoff used for dichotomization is illustrated in Figure 4 and Figure A4. There was a clear pattern of trade-off between sensitivity and specificity, with cutoffs at higher apparent exposure values producing classifiers that favored specificity over sensitivity and *vice versa*. Cutoffs in the center of the apparent exposure distribution produced values of sensitivity and specificity that were similar to each other. The curves for sensitivity and specificity cross below the mean for controls and above the mean for cases. The specificity is unstable for smaller cutoff values and the sensitivity is unstable for larger cutoff values. Sensitivity tended to be higher among cases and specificity higher among controls. The deviation from non-differential misclassification was most pronounced when dichotomization was done at the extremes of the exposure distribution. Furthermore, the degree of divergence from non-differential misclassification increased with the strength of the true exposure-outcome association as well as the magnitude of the measurement error variance. Receiver operator characteristic (ROC) curves for selected cutoffs (−3, −2, −1, 0, 1, 2, 3) are shown in Figure A5. In general, the ROC curves varied little by the cutoff used to dichotomize W_1_, particularly when the true association was weak.

## 4. Discussion 

As expected, the cutoff (s) chosen for categorizing continuous variables has a profound effect on the results of this simulation study. The two types of OR plots presented here allow us to gain insight into the shape of the underlying exposure-outcome relationship, particularly in scenarios where there is minimal measurement error. This builds on our previous work [1,4] when we did not know the true shape of the underlying relationship. A U-shaped curve when the measure of the exposure of interest was dichotomized indicated that the underlying relationship was linear. This may have particular implication for meta-analyses of studies with binary exposures and outcomes. Cutoffs at different exposure values measured with different degrees of accuracy across studies may manufacture U-shaped associations and other non-linearities where none may in fact exist. As measurement error increases, we lose the ability to infer the shape of the underlying exposure-response relationship if the exposure of interest is dichotomized. The plots used for five categories of W_1_ may be a good way to infer shape, even in the presence of larger measurement error. In practice, it may be sensible for a *post hoc* correction for measurement error (e.g., [22,23]) to be applied to the data before construction of the OR curve.

It is particularly important to investigate a wide range of cutoffs if there is a suspected inflection point as failure to include the true inflection point in the OR curve would likely lead to incorrect assumptions about the underlying shape of the exposure-response relationship. (Methods, such as those used by Hinkley [24] or Muggeo [25], may be used to identify such inflection points. However, we do not know how these operate with multiple sources of measurement error (in exposure and outcome) or under the realist conditions in ASD research that we simulated. Thus, we maintain that exploring the extent to which our approach is useful in addressing such question was justified, especially since our method is more easily implemented by researchers who do not have the support needed to implement more advanced statistical methods.) The number of categories and cutoffs are also important considerations as analyzing too many categories may lead to instability in the curve and incorrect inferences about the exposure-response relationship. If many categories are needed or desirable it is better to have a greater distance between cutoffs to obtain a clearer picture of the exposure-response relationship by minimizing cross-contamination of adjacent exposure groups. This simulation does not take into account random sampling error, the effects of which are well known. The use of smaller samples will lead to increasing instability, particularly in the tails of the OR curve. Our previous work [1] illustrated that in an OR curve for a large administrative dataset (n ~ 19,000), a relatively wide range of ORs was possible for what is suspected to be, at most, a modest association. Although we simulated a realistic scenario in autism research, model misspecification is an additional potential source of bias in this simulation and similar observational studies.

Use of non-parametric methods to model non-linearity is an attractive alternative to categorization [7]. But there are problems for which one is interested in the change in risk due to a change in exposure below a certain level (as is the case in environmental epidemiology and is the aim of regulatory exposure limits). Furthermore, our method does not make any assumptions that are otherwise involved in modeling exposure-response shapes, e.g., splines assume no measurement error. We in fact show that under ‘small’ measurement error our approach can yield insights into the true shape of the exposure-response association without invoking unfamiliar or hard to test assumptions, or complications involved in selection of the exact form of the spline to fit to the data (e.g., number of nodes and degree of smoothing are left to the investigator’s judgment and can be a source of uncertainty about just how much a given result is an artifact of selective reporting). Yet it is clear that measurement error leads to loss of features in the data and it is therefore perilous to infer any specific shape of the exposure-outcome association based on our method or any other method that does not correct for measurement error. Thus, we again reinforce the need to understand the nature of measurement error present in any particular analysis.

## 5. Conclusions

We presented results for a simple scenario in which there are no confounders and the theoretically optimal cutoffs for the other variables included in the regression models are known. The scenario presented here is common in autism research as well as other areas of epidemiology, in which sex and (gestational) age are included in the regression model even if they are not true confounders. We do not know how the presence of confounding may influence the OR curve and the inferences about the underlying shape of the exposure-response relationship but it is likely that it will be obscured. Our SAS code is available upon request for other researchers to apply to other scenarios. 

A discussion of the merits and limitations of categorization of continuous variables is outside the scope of this study and has been discussed in-depth elsewhere (e.g., Bennette and Vickers [6]). This study reinforced results of our previous work that demonstrated that although vastly different numerical estimates may be obtained with different cutoffs, presenting results for a wide range of cutoffs is feasible, provides valuable insight into the exposure-response relationship when measurement error is small, and may help prevent fishing for a desired study result [1,4]. We also demonstrate that noticeable differential exposure misclassification arises when measurement error is present in a dichotomized exposure estimate and the cutoff is at the extremes of the observed distribution. Thus, it is important to consider that in such settings, an investigator may incur bias of unpredictable direction despite attempting (as is often done) to reduce the impact of measurement error by separating truly and highly exposed subjects from the rest.

It is possible (and relatively straightforward) to present measures of association for a wide range of cutoffs used to dichotomize a continuous exposure. If only one such cutoff is presented, the choice of cutoff has profound effects on the study results. Except in the presence of large measurement error, the shape of the OR curve may provide insight into the true shape of the underlying exposure-disease relationship.

## List of Abbreviations

AOSI: Autism Observation Scale in Infants
ASD: autism spectrum disorders
EARLI: Early Autism Risk Longitudinal Investigation
OR: odds ratio
